# How Ants Drop Out: Ant Abundance on Tropical Mountains

**DOI:** 10.1371/journal.pone.0104030

**Published:** 2014-08-06

**Authors:** John T. Longino, Michael G. Branstetter, Robert K. Colwell

**Affiliations:** 1 Department of Biology, The University of Utah, Salt Lake City, Utah, United States of America; 2 Department of Entomology, National Museum of Natural History, Smithsonian Institution, Washington, D.C., United States of America; 3 Department of Ecology and Evolutionary Biology, University of Connecticut, Storrs, Connecticut, United States of America, and University of Colorado Museum of Natural History, Boulder, Colorado, United States of America; University of Guelph, Canada

## Abstract

In tropical wet forests, ants are a large proportion of the animal biomass, but the factors determining abundance are not well understood. We characterized ant abundance in the litter layer of 41 mature wet forest sites spread throughout Central America (Chiapas, Guatemala, Honduras, Nicaragua, and Costa Rica) and examined the impact of elevation (as a proxy for temperature) and community species richness. Sites were intentionally chosen to minimize variation in precipitation and seasonality. From sea level to 1500 m ant abundance very gradually declined, community richness declined more rapidly than abundance, and the local frequency of the locally most common species increased. These results suggest that within this elevational zone, density compensation is acting, maintaining high ant abundance as richness declines. In contrast, in sites above 1500 m, ant abundance dropped abruptly to much lower levels. Among these high montane sites, community richness explained much more of the variation in abundance than elevation, and there was no evidence of density compensation. The relative stability of abundance below 1500 m may be caused by opposing effects of temperature on productivity and metabolism. Lower temperatures may decrease productivity and thus the amount of food available for consumers, but slower metabolisms of consumers may allow maintenance of higher biomass at lower resource supply rates. Ant communities at these lower elevations may be highly interactive, the result of continuous habitat presence over geological time. High montane sites may be ephemeral in geological time, resulting in non-interactive communities dominated by historical and stochastic processes. Abundance in these sites may be determined by the number of species that manage to colonize and/or avoid extinction on mountaintops.

## Introduction

What determines the number of ants in 1 m^2^ of forest floor leaf litter? This disarmingly simple question is not easy to answer, because many processes potentially influence ant density: temperature effects on food availability [1]–[3], temperature effects on metabolism and foraging [3]–[5], litter depth effects on food availability and habitat space [6]–[9], moisture effects on food availability and foraging efficiency [10]–[13], seasonality effects on food and foraging [10] [1], species composition effects through species packing and niche partitioning [14], and availability of species in a regional species pool [15]. Ecologists have a long-standing interest in explaining ecological patterns at multiple spatial scales [4] [5] [15] [16], including the smallest scale relevant to particular groups of organisms. Small-scale abundance patterns in land plants have been extensively studied [17], but less attention has been paid to similar patterns in consumers. This study examines ant abundance at the 1 m^2^ scale in 41 Central American forests, across a broad range of elevations. We focus on temperature and species richness as primary explanatory variables.

Longino and Colwell [18] documented ant abundance on the Barva Transect, a continuously-forested elevational gradient in Costa Rica. Ant abundance was curvilinear as a function of elevation, changing relatively little from sea-level to 1500 m and then declining abruptly to near zero at 2000 m. In addition, the study yielded evidence of density compensation: at higher elevations there were fewer species, but more individuals per species, resulting in relatively stable total ant density even as diversity declined. However, the Barva Transect study covered a limited geographic scope, with just seven discrete sampling elevations on a single mountainside. Here, we aim to examine patterns of ant abundance with respect to elevation based on a much larger dataset (34 additional sites), over a larger spatial extent (Costa Rica to southern Mexico), and with a larger number of high-elevation sites. This more comprehensive dataset has revealed broadly similar patterns across all of Central America and documents previously unappreciated patterns of ant decline above 1500 m.

## Methods

Research sites were a combination of private land and state protected lands. Research and collecting permits were obtained from El Colegio de la Frontera Sur in Chiapas, Mexico; Universidad del Valle, Guatemala; Consejo Nacional de Areas Protegidas, Guatemala; Escuela Agrícola Panamericana Zamorano, Honduras; Instituto Nacional de Conservación y Desarrollo Forestal, Areas Protegidas y Vida Silvestre, Honduras; Fundación para La Autonomía y Desarrollo de la Costa Atlántica de Nicaragua; Ministerio del Ambiente y los Recursos Naturales, Nicaragua; Jesus Mountain Coffee Group, Nicaragua; The Organization for Tropical Studies, Costa Rica; and the Ministerio de Ambiente y Energía, Costa Rica. No protected species were sampled.

Like the original Barva Transect data, this larger dataset limited the number of contributing variables by sampling exclusively in closed-canopy evergreen wet forest, thus removing or at least greatly reducing effects of aridity, seasonality, and major habitat differences on ant abundance and diversity. We chose widely dispersed sites in Central America, aiming for a compromise between sites that were far enough apart to reduce effects of spatial autocorrelation, yet close enough to reduce effects of dispersal limitation. To focus on temperature and species richness as primary explanatory variables, we selected sites across a range of elevations from 0–2600 m. Because none of our sites were ever exposed to freezing temperatures, adaptations for frost-tolerance were not relevant.

**Figure 1:**
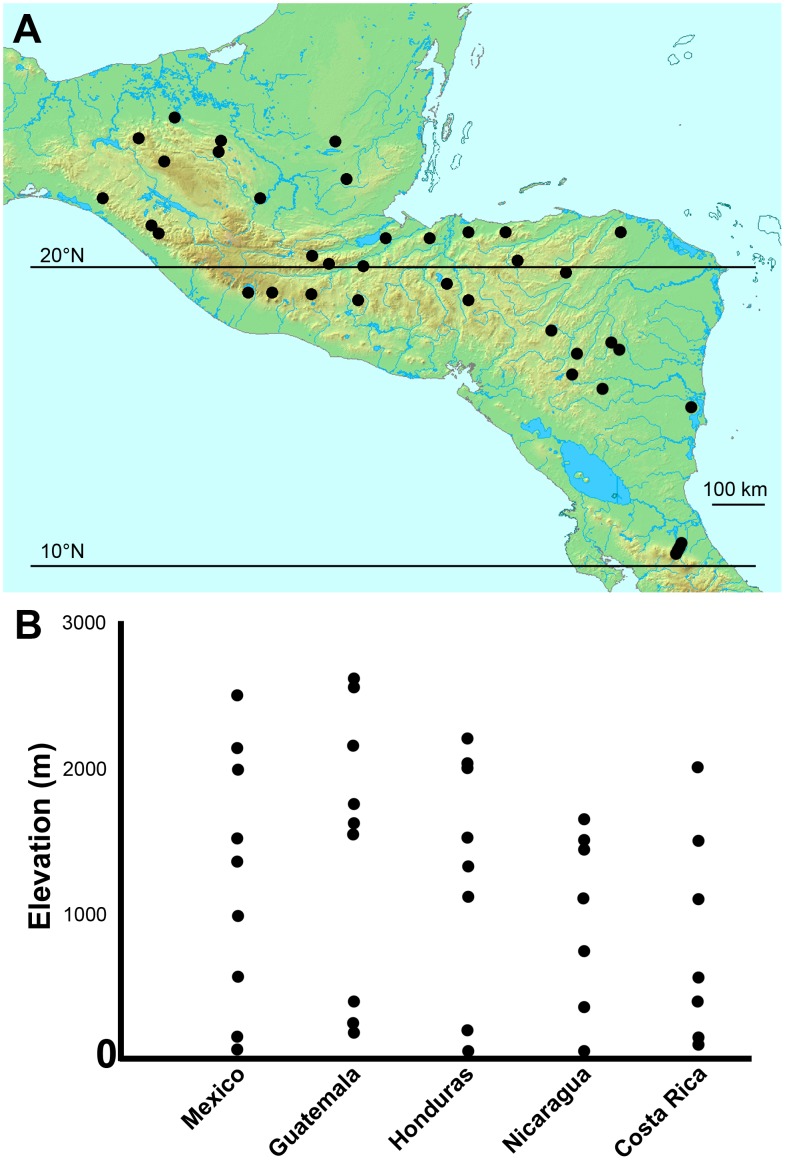
Geographic (A) and elevational (B) distribution of study sites.

Field sampling was carried out at 41 sites from Chiapas, Mexico, to Costa Rica (Fig. 1, Table 1). We used a subset (7 sites) of the data reported in Longino and Colwell [18], sampled from 2001 to 2007 as part of the Arthropods of La Selva project (ALAS, http://purl.oclc.org/ALAS) and Conservation International's Tropical Ecology Assessment and Monitoring project (TEAM, http://www.teamnetwork.org/en/). The remaining 34 sites were distributed in wet forest throughout Nicaragua, Honduras, Guatemala, and the Mexican state of Chiapas. These sites were part of the Leaf Litter Arthropods of MesoAmerica (LLAMA) project (https://sites.google.com/site/longinollama/) and were sampled from 2008 to 2011, following the same protocols used by the ALAS and TEAM projects for the Costa Rican sites.

**Table 1:** Sample sites for leaf litter ants in Central America.

Country	Locality	Latitude	Longitude	Elevation (m)
Mexico	La Sepultura	16.160	−93.605	1360
Mexico	El Triunfo low	15.721	−92.951	1520
Mexico	El Triunfo high	15.710	−92.929	2140
Mexico	Coapilla	17.176	−93.132	1990
Mexico	Huitepec	16.747	−92.490	2500
Mexico	Metzabok	17.127	−91.630	570
Mexico	Nahá	16.964	−91.593	985
Mexico	Salto de Agua	17.515	−92.296	70
Mexico	Playón de la Gloria	16.160	−90.901	160
Guatemala	Sierra de las Minas	15.084	−89.946	2560
Guatemala	Biotopo el Quetzal	15.212	−90.215	1750
Guatemala	La Unión	14.947	−89.276	1550
Guatemala	Montaña Chiclera	15.511	−88.861	195
Guatemala	Tikal	17.002	−89.717	250
Guatemala	Machaquilá	16.446	−89.550	400
Guatemala	Cerro Santiago	14.530	−90.149	2600
Guatemala	Cerro Carmona	14.536	−90.694	2150
Guatemala	Volcán Atitlán	14.549	−91.191	1625
Honduras	La Muralla	15.099	−86.741	1530
Honduras	Sierra de Agalta	14.949	−85.915	2020
Honduras	Cerro Comayagua	14.460	−87.545	2000
Honduras	Azul Meambar	14.871	−87.899	1120
Honduras	Guisayote	14.456	−89.069	2200
Honduras	Cusuco	15.487	−88.234	1330
Honduras	Río Platano	15.664	−84.858	60
Honduras	Lancetilla	15.764	−87.457	30
Honduras	Pico Bonito	15.694	−86.863	200
Nicaragua	Musún	12.961	−85.232	750
Nicaragua	Saslaya low	13.769	−84.984	360
Nicaragua	Saslaya high	13.772	−85.012	1110
Nicaragua	Datanlí El Diablo	13.110	−85.868	1440
Nicaragua	Kilambé	13.570	−85.697	1500
Nicaragua	Cerro Jesús	13.982	−86.189	1650
Nicaragua	Wawashang	12.672	−83.716	30
Costa Rica	Barva Transect	10.416	−84.020	120
Costa Rica	Barva Transect	10.404	−84.039	150
Costa Rica	Barva Transect	10.345	−84.058	400
Costa Rica	Barva Transect	10.317	−84.049	570
Costa Rica	Barva Transect	10.267	−84.083	1100
Costa Rica	Barva Transect	10.236	−84.118	1500
Costa Rica	Barva Transect	10.183	−84.117	2000

Sampling was carried out during the dry season for the ALAS samples, throughout the year for TEAM samples, and during the dry to wet season transition for LLAMA samples. Site histories and disturbance levels varied among sites, but most sampling was performed in relatively mature forest patches. The highlands of Honduras, Guatemala, and Chiapas often have island-like areas of diverse mesophyl cloud forest surrounded by pine or pine/oak forests; LLAMA sampling in these areas was always in the mesophyl cloud forest. Thus, other than elevational differences, the sites were substantially uniform with respect to physiognomy and forest structure.

The sampling unit was a “miniWinkler” sample of arthropods extracted from a 1 m^2^ forest floor quadrat, following the methods of Fisher [19]. Litter in a quadrat (including any vegetation and suspended organic matter immediately above the quadrat) was chopped with a machete, gathered into a sifter, and shaken vigorously. Sifting continued until all litter in the plot was sifted or a maximum of 6 L of siftate was obtained. In the latter case, material from different parts of the plot was subsampled. Although not quantified, the proportion of quadrats that required subsampling was low and did not appear to vary with elevation. Siftate was transferred to cloth sacks and moved to a laboratory or shelter, where each sample was suspended in an individual Winkler extractor for three days. Falling arthropods were collected into 95% ethanol. For the three ALAS sites on the Barva Transect and the 34 LLAMA sites, miniWinkler samples were distributed in two haphazardly-placed transects of 50 samples, with 5 m spacing between samples. For the four TEAM sites on the Barva Transect, samples were distributed in 10 transects of 10 samples each, with 10 m spacing between samples. Total sample size was 4099 miniWinkler samples (one sample was lost).

All ants were separated from samples by project staff. The total number of workers was recorded for each sample, as a measure of overall ant abundance. Only a subset of the ants in each sample was identified to species; certain taxa were excluded due to taxonomic impediments (genera that are very difficult to sort to species), habitat edge effects (arboreal species that occasionally occur in Winkler samples), and pest ants that were likely contaminants. Genera excluded due to taxonomic difficulty were *Brachymyrmex* (except the distinctive litter species *B. cavernicola*), *Hypoponera* (except the distinctive species *H. nitidula* and *H. parva*), *Nylanderia*, *Solenopsis* (except *S. geminata*), and *Tapinoma*. Excluded arboreal taxa were *Azteca*, *Camponotus*, *Cephalotes*, *Dolichoderus*, *Myrmelachista*, *Procryptocerus*, *Pseudomyrmex*, and *Xenomyrmex*. Excluded pest ants were *Cardiocondyla*, *Monomorium*, *Paratrechina longicornis*, and *Tetramorium bicarinatum*. Identifications were carried out by Branstetter (*Stenamma*) and Longino (remaining taxa). Voucher specimens were deposited in regional collections in Costa Rica, Honduras, Guatemala, and Chiapas; in the Longino research collection at The University of Utah; and in the Branstetter research collection. The personal research collections of Longino and Branstetter will ultimately be deposited in major institutional collections. All specimen data are posted on AntWeb (www.AntWeb.org).

Bioclimatic variables were extracted for all 41 sites from WorldClim global climate data ([20]; http://www.worldclim.org, Version 1.4, release 3; accessed 11 July 2012) for the latitude and longitude coordinates in Table 1, using “current values” (averages from 1950–2000). Four variables were examined: mean annual temperature, minimum temperature of the coldest month, annual precipitation, and precipitation seasonality (coefficient of variation of monthly averages) (WorldClim variables BIO1, BIO6, BIO12, and BIO15, respectively). Minimum temperature of the coldest month and precipitation seasonality were highly collinear with mean annual temperature and annual precipitation, respectively, and are not considered further. Annual precipitation varied from 1230–4400 mm across sites, but explained negligible variance in ant abundance, suggesting that our selection of wet evergreen sites successfully removed moisture as a contributing variable. Mean annual temperature and elevation were highly collinear (r^2^ = 0.96). Often relationships were “cleaner” (higher r^2^) when regressed on elevation rather than on WorldClim temperature. Elevation may be a better proxy for average temperature at a site. The adiabatic lapse rate insures a constancy of elevation - temperature relationship within a region [21] that may match or exceed the accuracy of WorldClim estimates. Thus, elevation was used as a proxy for temperature in this study.

Within sites, number of workers was approximately normal following log(*n*+1) transformation. Average worker abundance, the main dependent variable in this study, was the mean of the 100 log(*n*+1)-transformed values for a site.

Species richness was the number of species in the 100 miniWinkler samples, using only the subset of identified taxa (a “taxonomic sufficiency” approach; see [22]). Observed species richness was used instead of rarefaction to a common sample coverage (*sensu* [23]) because sample coverage was above 0.93 in all cases and the observed richnesses were highly collinear with rarefied richness. Important to our analyses was the assumption that the relationship between included and excluded taxa was unbiased with respect to elevation. Examination of the seven Barva Transect sites, for which all ant species were sorted to species [18], allowed us to evaluate the impact of using a subset of taxa. Subset species richness varied from 61–89% of total species richness across the seven sites, but the proportion was unrelated to elevation (r^2^ = 0.1).

Average worker abundance was examined as a function of elevation (a proxy for temperature), species richness, and their interaction using linear models (“lm” and “AIC” functions) in R v. 2.15.1 [24]. We report AICc, which corrects AIC for small sample size effects. At each site, we determined which one of the local species was the most abundant at that site, and computed its local frequency of occurrence in the 100 miniWinkler samples. This “frequency of the most common species” was examined as a function of species richness. An index of abundance per species was calculated as the average worker abundance divided by the total number of species in the 100 miniWinkler samples. It is an index because worker abundance was based on all ants and the species count was based on a subset of ant species (excluding taxonomically problematic genera). Density compensation was inferred by (1) a weak relationship between abundance and species richness, (2) a negative relationship between species richness and the frequency of the most common species, and (3) a positive relationship between abundance per species and elevation.

## Results

**Figure 2:**
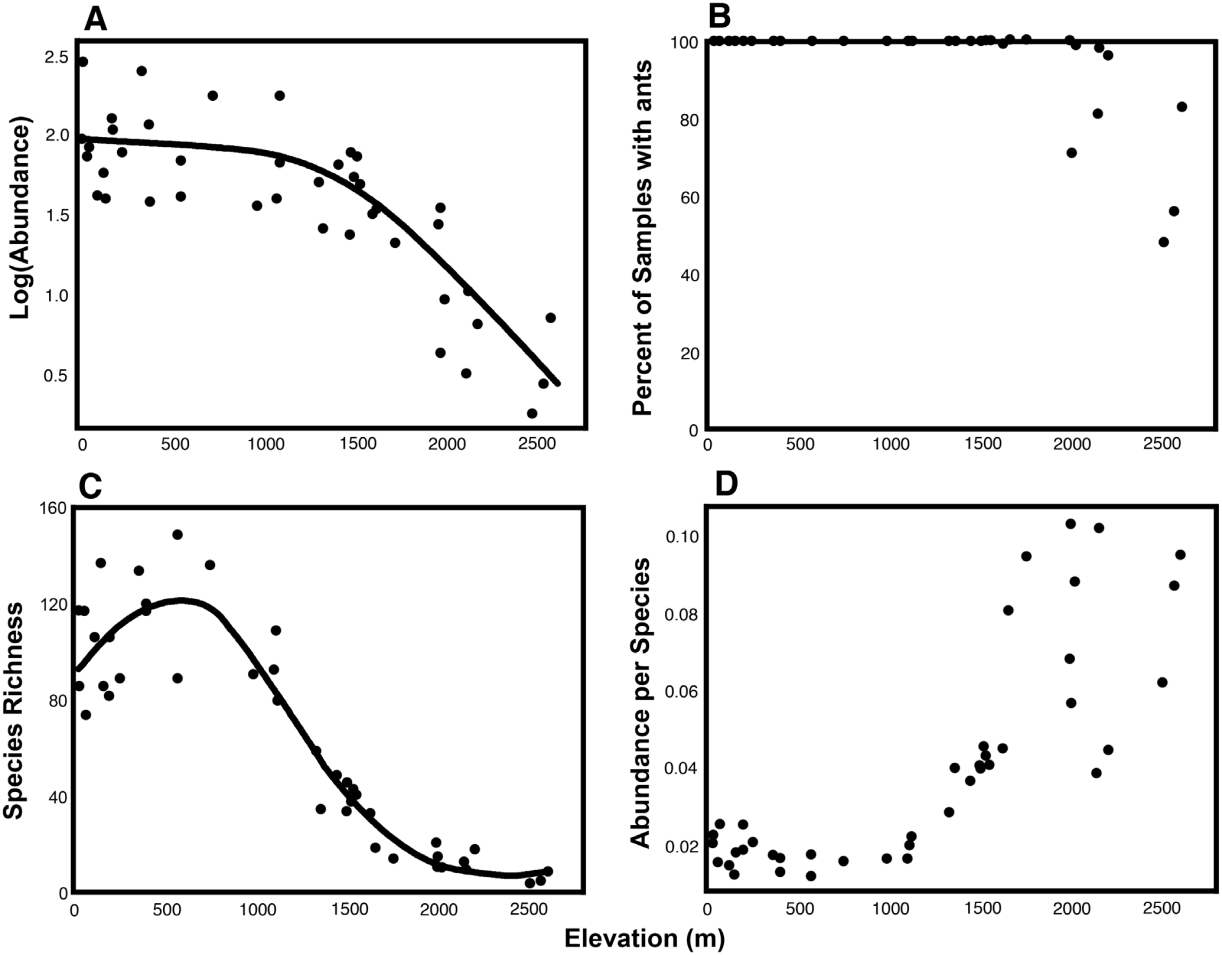
Ant abundance and richness variables as a function of elevation among 41 Central American wet forest sites. A. Log(Abundance) = average of 100 log[*n*+1] values where *n* = number of workers in 1 m^2^. B. Percent of samples containing ants. C. Species Richness = number of species in 100 samples (taxon subset; see text). D. Abundance per species = Abundance as defined in panel A/Richness as defined in panel C. LOESS curves are included for A and C, using default parameters in R 3.0.2.

Average worker abundance very gradually declined from sea level to 1500 m and very steeply declined above 1500 m, with ants becoming extremely rare at the highest sampled sites, 2500–2600 m (Fig. 2A). In all lowland and mid-elevation sites, all or nearly all samples contained ants; whereas in high montane sites, >1500 m, samples showed variable occupancy, with up to 50% lacking ants (Fig. 2B). Species richness showed high values from 0–1000 m, with a peak around 500 m, a sharp drop from 1000–2000 m, and a more gradual decline above 2000 m (Fig. 2C). Abundance per species was flat to 1000 m elevation, rose linearly between 1000 and 1500 m, but then “exploded” above 1500 m, becoming highly variable and unrelated to elevation (Fig. 2D).

**Figure 3:**
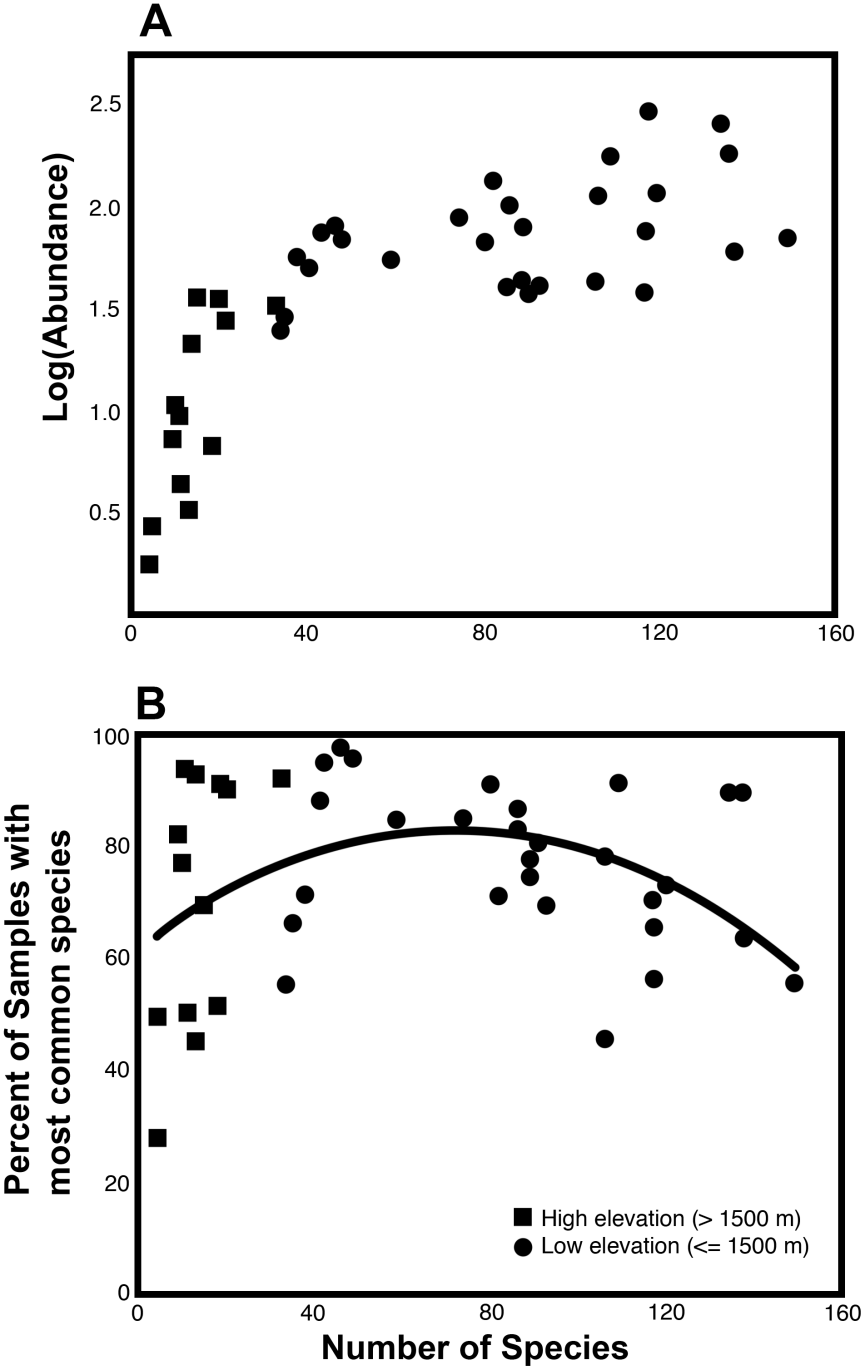
Ant abundance variables as a function of species richness among 41 Central American wet forest sites. Number of Species = number in 100 samples (taxon subset; see text). Symbols differentiate low and high elevation sites (elevation rounded to nearest 100 m). A. Log(Abundance) = average of 100 log[*n*+1] values where *n* = number of workers in 1 m^2^. B. Percent of samples containing the single most abundant species at each site. The curve shows the fitted quadratic model.

The effect of species richness on abundance varied with elevation, as shown by a strong interaction between elevation and richness in linear models (Table 2). Abundance showed a weak positive relationship to species richness in lowland rainforests and mid-elevation cloud forests, and a very strong positive relationship among the high montane forest sites (Fig. 3A). When the ten sites above 1950 m elevation were analyzed separately, the only significant explanatory variable was species richness (Table 2).

**Table 2:** Linear models for effects of elevation (as a proxy for temperature) and species richness on average ant worker abundance at 41 Central American forest sites.

Model	I	R	E	R*E	*p*	r^2^	AICc
*All sites*							
Richness	1.01	0.0089			***	0.56	34.42
Elevation	2.16		−0.0005		***	0.59	31.81
Richness + Elevation	1.68	0.0039	−0.0003		***	0.61	31.53
Richness + Elevation + (Richness*Elevation)	2.24	−0.0032	−0.0007	7.76E-6	***	0.72	18.57
*Ten highest sites*							
Richness	0.19	0.0563			*	0.45	12.90
Elevation					n.s.	0.28	15.47
Richness + Elevation					n.s.	0.39	18.44

Species richness is the number of species in 100 samples, using a taxon subset of identified ants (see text). Coefficient estimates have column headings I, R, E, R*E for intercept, richness, elevation, and interaction, respectively.

The percent of samples containing the single most abundant species at a site varied with species richness (Fig. 3B). A linear model of percent of samples as a function of number of species was not significant, whereas a quadratic model was significant (p<0.05, r^2^ = 0.10) and with lower AIC value. The same analysis performed on arcsin(squareroot)-transformed frequencies was non-significant (p<0.065 for quadratic model) but retained the same trend. The results suggest that among high diversity sites (lowland rainforest and cloud forest), the common species become more common if overall species diversity is lower. In contrast, among high montane sites, the common species decrease in abundance as overall diversity declines.

## Discussion

A robust pattern of ant abundance in evergreen wet forests prevails throughout Central America. The average 1 m^2^ patch of leaf litter and dead wood on the forest floor is filled with a similar number of ant workers, from steamy lowland rainforest at sea level to cool cloud forest at 1500 m. Above 1500 m there is an abrupt change, and abundance declines to near 0 above 2500 m. This pattern was revealed on one mountainside in Costa Rica [18] and is now shown to be remarkably consistent on similar mountains throughout Central America. The pattern persists whether in continuously forested elevational gradients like the Barva Transect in Costa Rica or in small forest patches surrounded by pine forest, coffee farms, or pastures in Nicaragua, Honduras, Guatemala, and southern Mexico. What factors might explain this pervasive pattern?

The energy limitation hypothesis posits that, for organisms of similar size, abundance of consumers is controlled by available energy, and as a result, abundance will be positively correlated with measures of productivity [25] [16] [2]. Ants provide empirical support for this relationship at a continental scale [1] [2] [14] but not always at local scales [4].

The lack of a productivity - abundance relationship at small scales may be explained by the fact that both producers and consumers are affected by temperature. Brown et al. [3] outlined a metabolic theory of ecology, describing the pervasive effects of metabolism on many traits of ecological communities. They predicted that population density or carrying capacity, *K*, would follow the relationship:
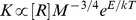
where *R* is a resource supply rate, *M* is body mass, and the last term is the Boltzmann factor (*E* is the activation energy of metabolic reactions, *k* is Boltzmann's constant, and *T* is absolute temperature in degrees Kelvin). Thus carrying capacity *K* should increase with supply rate *R* (the energy limitation hypothesis), decrease with *M*, and, somewhat paradoxically, decrease with *T* (at least for plants and ectotherms, where body temperature tracks environmental temperature; [26]). As *T* increases, all else being equal, metabolism will increase, as will energetic turnover rate, resulting in lower standing biomass. For consumers, the resource supply rate is NPP, which is affected by many factors including stoichiometry, nutrient availability, water, light, and temperature [27]. But NPP is often mainly temperature-limited in mesic sites [27] [1] such as those studied here. In the wet tropics, resource supply rates of all kinds (solar radiation, chemical nutrient availability, water) may be similar across elevations, with only *T* varying. If plant metabolism is limited by the same Boltzmann factor as consumer metabolism, NPP and temperature effects will act in opposition, so that *K* may become relatively insensitive to NPP and *T*. NPP in wet forests is predicted to decline at colder temperatures (higher elevations), and thus the amount of food available for consumers like ants will also decline. However, the ants at those cooler temperatures may lead lives at a slower tempo, keeping the same biomass as lowland ants, but with a lower rate of energy use (see also [28]).

Density compensation patterns similar to those observed on the Barva Transect [18] occurred throughout Central America. Species richness is lower in cloud forests than in lowland rainforests, but ants nevertheless fill up the environment. Average abundance remains high and the most abundant species achieve higher frequencies than they do in lowland rainforests.

An alternative hypothesis we have not fully assessed is that the relative stability of ant abundance is an artifact of an increase in litter depth with elevation. Litter depth and organic matter content increase with elevation in tropical forests [29], and positive correlations of litter depth and ant abundance have been observed in other studies [7] [9]. We may be seeing more ants at higher elevations because we are sampling a greater volume of habitat, whereas ant density per volume is actually decreasing. We cannot fully rule out a litter volume effect, but our sampling method limited the potential impact by imposing a maximum sample volume of 6 L (Methods). When litter volumes were high, only a portion of the litter within a 1 m^2^ plot was sifted.

Starting above 1500 m, very different processes are occurring. In high montane forests, abundance sharply declines and is closely related to species richness. There is no evidence of density compensation. A partial explanation for the shift above 1500 m may be the appearance, beginning at about that elevation, of areas unsuitable for any ant species. Within a site, shading causes spatial variation in the thermal environment. Individual points on the forest floor often vary dramatically in the amount of direct solar radiation they receive. One possibility is that at 1500 m and below, all sites are inhabitable by at least one species of ant in the habitat, while above this elevation an increasing proportion of the forest floor is too cold for any ant species to survive. Metabolic theory predicts a stable abundance within habitable space. The abrupt shift to declining worker abundance above 1500 m may simply reflect an increasing proportion of ant-free space in the area sampled.

However, this mechanism by itself does not explain the relationship of abundance to species richness in high montane forests. If habitable space were only a function of temperature, independent of the regional species pool, one would expect abundance to be more closely related to elevation than to species richness. The regional species pool of highland specialists may exhibit a range of cold tolerances, affecting the amount of habitable space. Historical effects of vicariance, dispersal limitation, and age of high mountains may determine the species pool, which in turn determines the amount of habitable space. Hypotheses regarding the effects of ant-free space and regional species pools could be tested with fine-scale microhabitat measurements in montane sites, to see if ant species are tracking particular thermal environments and if montane species vary in cold tolerances.

The patterns observed here are emergent properties that arise despite shifting community composition. Longino and Colwell [18] showed sharp transitions in community composition among lowland, cloud forest, and high montane forests. Although not analyzed in detail here, these patterns hold throughout Central America. The lowland rainforest communities show high species and genus-level diversity, with particularly high numbers of species in the hyperdiverse genera *Pheidole* and *Strumigenys*. Common species in the high montane sites are all montane specialists that do not occur in the lowlands. They include a few species in the large genera *Pheidole* and *Strumigenys*, but Central American high montane sites are often dominated by species in the genera *Adelomyrmex* and *Stenamma*. These two genera have clearly diversified and become abundant in the mountains of Central America and are less diverse and less abundant in the lowlands [30] [31]. Mid-elevation cloud forests are intermediate with respect to representation of lowland and highland lineages, but at the species level are distinct from both. There is also strong species turnover latitudinally, with little overlap in species composition between Costa Rica and southern Mexico (Longino et al., unpublished data). More quantitative investigation of phylogenetic structure across these assemblages is clearly needed (e.g., [32]).

Long-term equilibrial processes may be shaping the lowland and cloud forest communities on these gradients. Even with Pleistocene climate fluctuations and large elevational shifts of climate zones, lowland and cloud forest habitats probably remained broadly available and within easy dispersal distance on tropical mountain slopes, allowing community structure to be shaped by competitive interactions over long periods of contact. Is the lower diversity we found in cloud forest habitats driven by equilibrial processes? In ecological time, by the paradoxical prediction of the metabolic theory, does the lower energy availability result in higher minimum population sizes, thereby limiting diversity? In evolutionary time, does lower energy availability increase extinction rates relative to speciation rates? These hypotheses predict stable abundance and diversity relationships over long periods of time. Alternatively, are the patterns stable in ecological time but not in evolutionary time, such that the difference between lowland and cloud forest diversity is due to a lowland head-start during the Tertiary? Are cloud forest faunas younger, the result of planetary cooling during the Pleistocene and the creation of extensive cloud forest habitat? These questions so far remain unanswered.

In contrast to lowland and cloud forest communities, non-equilibrial processes may dominate the high montane communities. A small number of species occur at the highest elevations, and they are constrained to those elevations. Abundance relationships are highly variable and there is no evidence of density compensation at these high elevations. The strongest predictor of ant abundance is the number of species present. Variation in the number of species in these high montane sites is not related to elevation, and thus may be more influenced by stochastic processes of extinction and colonization. High montane sites may be evolutionarily more ephemeral and more island-like, routinely appearing and disappearing through the vagaries of orogeny and, on a shorter time scale, by Pleistocene glacial cycles. In these highest sites, age of habitat and dispersal limitation may be the dominant factors influencing diversity and abundance relationships.
